# An uncommon manifestation of paraneoplastic cerebellar degeneration in a patient with high grade urothelial, carcinoma with squamous differentiation: A case report and literature review

**DOI:** 10.1186/s12885-016-2349-3

**Published:** 2016-05-21

**Authors:** Yaofeng Zhu, Shouzhen Chen, Songyu Chen, Jing Song, Fan Chen, Hu Guo, Zhenhua Shang, Yong Wang, Changkuo Zhou, Benkang Shi

**Affiliations:** Department of Urology, Qilu Hospital of Shandong University, Wenhua Xi Road, Jinan, Shandong Province People’s Republic of China; Department of Neurosurgery, Shanghai tenth people’s Hospital, Tongji University, Yanchang Zhong Road, Shanghai, People’s Republic of China; Shandong University School of Medicine, Wenhua Xi Road, Jinan, Shandong Province People’s Republic of China

**Keywords:** Paraneoplastic cerebellar degeneration, Bladder cancer, High grade urothelial, Carcinoma, Squamous differentiation

## Abstract

**Background:**

Paraneoplastic neurological syndromes (PNS) are rare disorders associated with malignant tumours, which are triggered by autoimmune reactions. Paraneoplastic cerebellar degeneration (PCD) is the PNS type most commonly associated with ovarian and breast cancer. Two bladder cancers manifesting in PCD were previously reported. However, the cancers in these cases had poor outcomes.

**Case presentation:**

Here, we present a 68-year old man with history of high-grade papillary urothelial carcinoma of the bladder. The patient suffered from persistent cerebellar ataxia accompanied by bladder cancer recurrence five months after transurethral resection of the bladder tumour (TURBt). Laboratory screening for the specific antibodies of paraneoplastic neurological syndromes revealed no positive results. Symptoms were not remitted after a 7-day-course of high-dose glucocorticoid therapy. To our surprise, the patient recovered fully after laparoscopic radical cystectomy. Postoperative pathology revealed that surgical specimens were urothelial carcinoma *in situ* (CIS) and squamous cell carcinoma of the bladder. The patient remained asymptomatic and there was no evidence of recurrence after the followup period of 11 months.

**Conclusion:**

To our knowledge, this is the third report of PCD in a patient with bladder cancer. This case showed that tumour resection cured the PCD. To assist clinical evaluation and management, literature regarding basic PNS characteristics and bladder cancers was reviewed.

## Background

The bladder cancer is the 11th most common site of cancer diagnosis and the 14th leading cause of cancer-related deaths in the world [1]. The 2004 WHO/ISUP system classifies papillary urothelial neoplasms into four types: papilloma, papillary urothelial neoplasm of low malignant potential, low-grade papillary urothelial carcinoma, and high-grade papillary urothelial carcinoma [2]. The high-grade papillary urothelial carcinoma was associated with the high recurrence rate. The main clinical treatment for non-muscle-invasive bladder cancer is transurethral resection of Ta and T1 bladder tumours [3]. However, carcinoma *in situ* (CIS) is a high-grade, flat, non-invasive urothelial form. This carcinoma is often multifocal and progresses to muscle-invasive disease. Therefore, endoscopic procedures alone are insufficient for CIS treatment. Either intravesical bacillus Calmette-Guérin (BCG) instillations or radical cystectomies (RC) are applied [4, 5]. Several studies have revealed excellent outcomes after immediate RC for CIS [6].

Paraneoplastic neurological syndromes (PNS) are rare disorders associated with malignant tumours that are not directly caused by tumour invasion and metastasis. The autoimmune response is elicited by the ectopic expression of neural antigens in neoplastic tissues, which eventually attack the nervous system of PNS patients [7]. PNS incidence is estimated to be 0.5–1 %, varying by cancer type [8, 9]. Paraneoplastic cerebellar degeneration (PCD) is the type of PNS most commonly associated with ovarian and breast cancer [9]. PCD is characterized by subacute cerebellar ataxia within 12 weeks and subsequent cerebellar atrophy [10]. Bladder cancers presenting with PCD have rarely been reported. Greenlee JE [11] and Tetsuka S [12] reported PCD and its causal antibody in bladder cancer patients. Here, we discuss a patient with urothelial CIS of the bladder who suffered from PCD. Unlike two previous cases, we found no specific antibody causing PCD. Literature concerning unusual cases are reviewed.

## Case presentation

A 68-year-old man was admitted to the Qilu Hospital of Shandong University on November 17, 2014 for urinary irritation, dysuria and haematuria. The patient’s condition was good, and weight loss, night sweats and recent fever did not present. Urinalysis showed red and white blood cell counts of 61.4/μL and 313.8/μL, respectively. Cystoscopy showed scattered cauliflower-like neoplasms located at 11°-1° of the narrow bladder neck, with a maximum diameter of 1.5 cm. A biopsy was then conducted and the pathological examination revealed that the neoplasms were high-grade papillary urothelial carcinoma of the bladder. CT scanning showed a thickened anterior bladder wall, without enlarged pelvic lymph nodes. Transurethral resection of the bladder tumour (TURBt) was conducted after a definitive diagnosis was made. Several villous tumours were found on the neck of the bladder during the operation. The postoperative pathological examination showed that the surgical specimens were high-grade papillary urothelial carcinoma accompanied by differentiated squamous cell carcinoma. This carcinoma also involved the prostate. Haematuria and dysuria symptoms disappeared after TURBt. Therefore, the patient’s family refused further treatment and the patient was discharged.

The patient was referred to our hospital again on March 27, 2015 because of vomiting, progressive gait imbalance and Haematuria for the previous five days. Neurological examinations revealed ataxic gait and bilateral coarse nystagmus. Slight dysmetria was confirmed by finger-to-nose and heel-knee-shin testing. Haematuria led to the performance of a urinalysis showing red and white blood cell counts of 554/μL and 6748/μL, respectively. Further cystoscopy revealed that bladder mucosa located at 11°-1° of the bladder neck suffered hyperaemia, edema, and erosion of the asperous surface. The biopsy showed a high-grade papillary urothelial carcinoma and squamous differentiation.

**Fig. 1:**
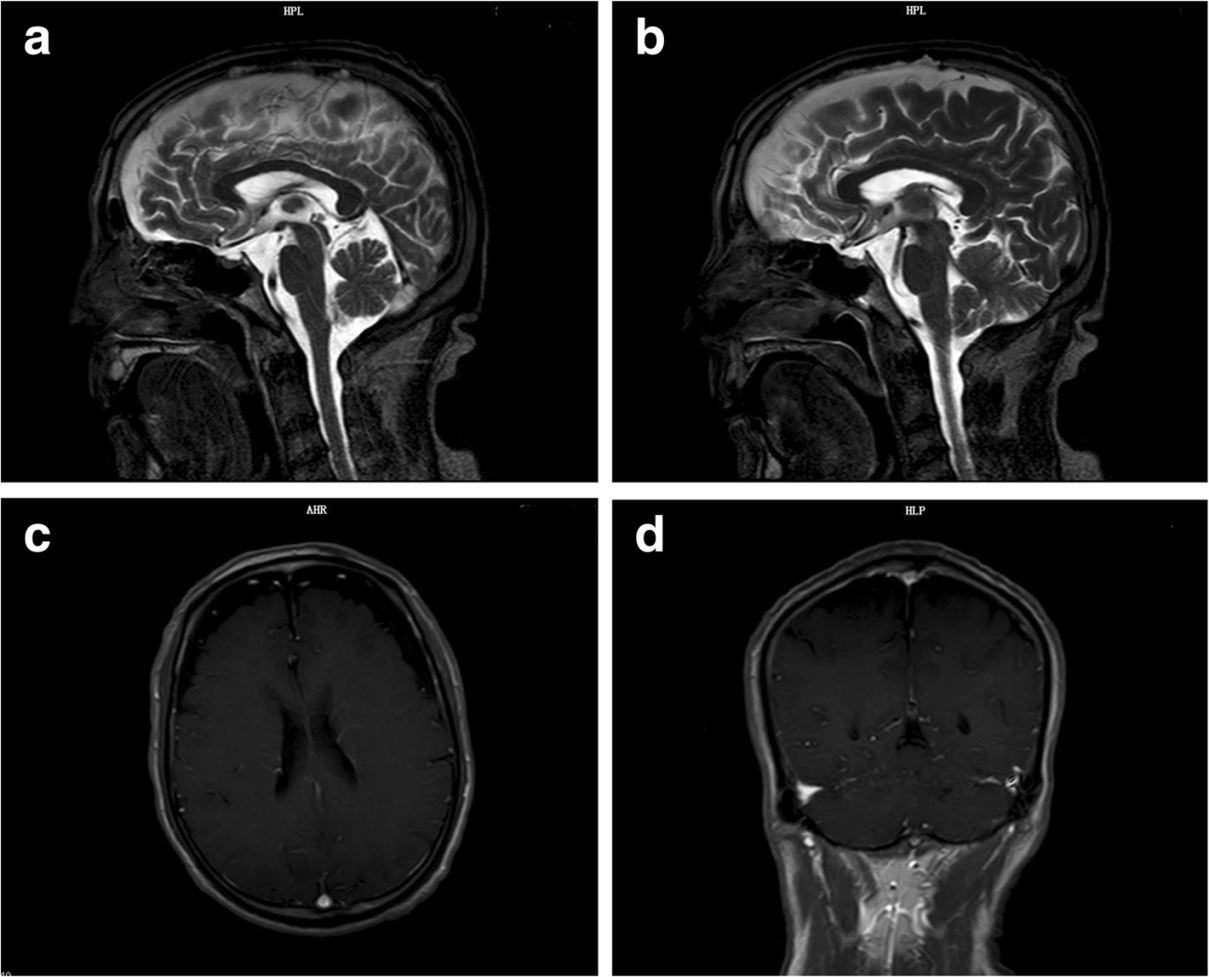
Magnetic resonance imaging (MRI) of the brain. **a** and **b**, the T2WI sagittal scan showed no obvious morphological change of the cerebellum; **c** and **d**, the enhanced-scanning MRI revealed no sign of brain metastases from bladder cancer

**Fig. 2:**
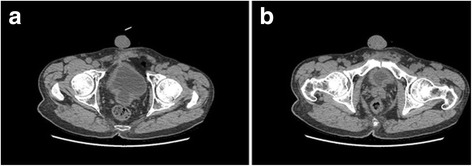
Computed tomography (CT) of the abdomen and pelvis. A, the bladder wall was thickened; B, the neck of bladder was thicker than the bladder wall

Further laboratory and radiographic evaluations were conducted to clarify the relationship between bladder cancer recurrence and emerging cerebellar ataxia. A brain MRI was performed to exclude brain metastases from bladder cancer (Fig. 1c, d). The MRI revealed no obvious morphological change of the cerebellum (Fig. 1 a, b). The abdominal and pelvic CT scans revealed that the bladder wall was thickened, especially at the neck (Fig. 2). The above radiographic findings suggest little possibility exists that nervous symptoms were triggered by brain metastases from bladder cancer.

Further laboratory examinations were conducted to validate the cause of neurological symptoms. Nine common types of paraneoplastic antibodies were all negative in the serum and cerebrospinal fluid (CSF). Details of the paraneoplastic antibodies are shown in Table 1. The CSF examination revealed an elevated level of protein (58.6 mg/dl, normal: 15–45 mg/dl), IgG (39.5 mg/L, normal < 34 mg/L), and albumin (391 mg/L, normal < 350 mg/L). Pandy's test of CSF was positive, while CSF cell counts were normal. Tumour markers showed that squamous cell cancer antigen (SCC) and non-small cell lung cancer antigen (CYFRA21-1) serum concentrations were elevated (SCC: 6 ng/ml, normal: 0.1–0.3 ng/ml; CYFRA21-1: 3.5 ng/ml, normal < 1.5 ng/ml). The thyroglobulin serum concentration was low (<0.04 ng/ml, normal: 1.4-78 ng/ml). The examination of rheumatic antigens revealed no positive results. The electroencephalogram revealed no epileptogenic activity.

**Table 1:** Examination Results of Paraneoplastic Antibodies

Type of antibodies: Ig G	
Antibody Description	Results
Anti-Amphiphysin	(−)
Anti-CV2.1	(−)
Anti-PNMA2/Ta	(−)
Anti-Ri	(−)
Anti-Yo	(−)
Anti-Hu	(−)
Anti-Recoverin	(−)
Anti-SOX1	(−)
Anti-Titin	(−)

(−) = negative

**Fig. 3:**
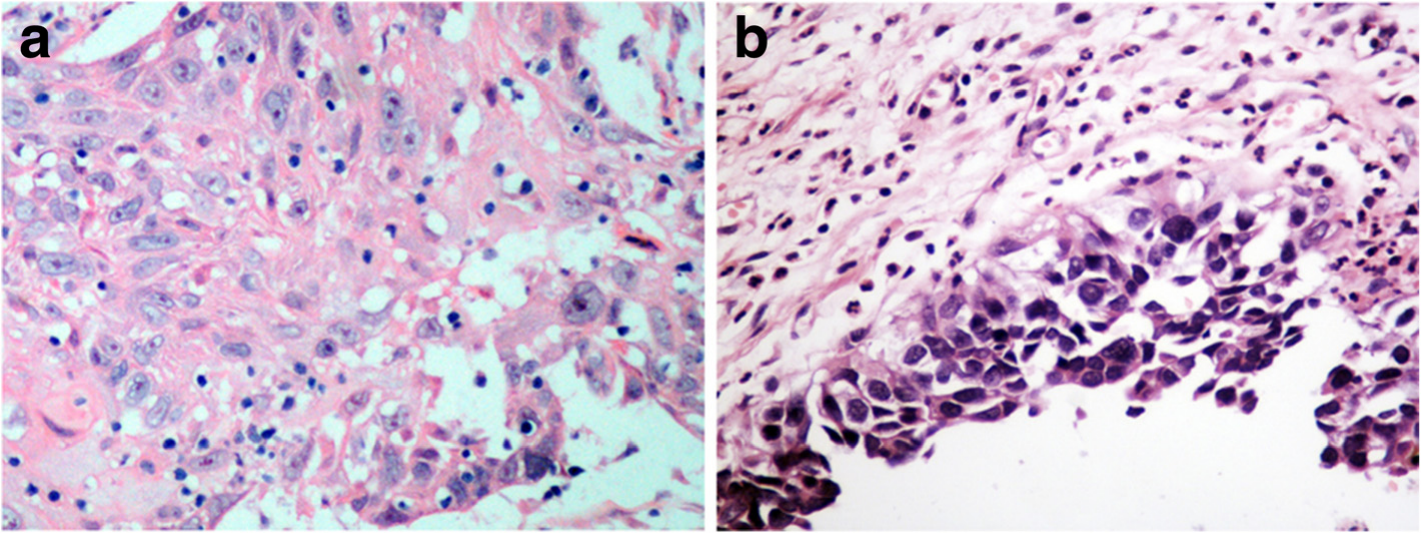
HE staining of bladder cancer tissues. **a**, HE staining showed irregular squamous cell carcinoma dispersed in the bladder mucosa. **b**, HE staining showed the urothelial CIS

Based on these findings, we suspected that the patient suffered from PCD. The patient initially received a 5-day course of methylprednisolone (500 mg intravenously daily) without significant clinical improvement. Considering the first postoperative pathological examination revealed the bladder cancer involved the prostate and recurred, the patient underwent laparoscopic radical cystectomy (LRC). The postoperative pathological examination revealed that the surgically removed bladder was urothelial CIS and well-differentiated squamous cell carcinoma (Fig. 3). This finding was in accordance with the changes in serum SCC and CYFRA21-1. The squamous cell carcinoma was dispersed in the bladder mucosa (Fig. 3a) and accounted for approximately 5 % of all carcinomas. Neurological symptoms disappeared, and basic daily living activities were obviously improved, according to Barthel score (Table 2), after LRC. The patient remained asymptomatic and there was no evidence of recurrence after the followup period of 11 months.

**Table 2:** Barthel Index of Activities of Daily Living Before and After LRC

Activities	Independent	Needs help	Unable	Before LRC	After LRC
Feeding	10	5	0	5	10
Transfer	15	5 or 10	0	0	10
Grooming	5	0	/	0	5
Toilet use	10	5	0	5	10
Bathing	5	0	/	0	5
Dressing	10	5	0	5	10
Mobility	15	5 or 10	0	0	10
Stairs	10	5	0	0	5
Bowels	10	5	0	10	10
Bladder	10	5	0	10	10
Total Score	100			35	85

## Discussion

PNS was initially defined as the presence of cancer and the exclusion of other known causes of the neurological symptoms. Examples of such causes include metastasis, coagulopathy, infections, metabolic or nutritional disturbances, and treatment-induced neurotoxicity [10, 13]. The exact pathogenesis of most PNS is unknown. However, neuronal antigens expressed by cancers could activate the immune system. Moreover, many antineuronal antibodies generated by the activated immune system attack the nervous system [7]. Antineuronal antibodies can affect different nervous system regions, and multiple clinical manifestations can present in PNS. When the cerebellum is affected, the rapid development of severe cerebellar ataxia is caused by extensive Purkinje neurons loss, defined as PCD.

According to the PNS Euronetwork recommended diagnostic framework in 2004 [13], the following criteria are required to define PCD: (1) severe pancerebellar syndrome developing in less than 12 weeks; (2) no radiographic evidence of cerebellar atrophy other than that expected by the patient’s age; and (3) the significant interference of symptoms caused by PCD with basic activities of daily living. Neurological syndromes developed in five days and the MR examnation excluded cerebellar atrophy in the present case. The Barthel activities of daily living Index was 35 after the onset of the disease, indicating a severe effect. All the characteristics in the present case met PCD diagnostic criteria. Other possible reasons for neurological syndromes were excluded. The neurological syndromes associated with bladder cancer could be clinically diagnosed as PCD.

PNS is commonly associated with ovarian cancer, breast cancer, SCLC, or Hodgkin’s disease. Few reports have associated PNS with bladder cancer. We retrospectively reviewed English literature regarding PNS and bladder cancers. We used PubMed, Medscape and EMBASE to search for studies on this topic, yielding eight articles. Only two reported a relationship between PCD and bladder cancer. Therefore, this study is the third to describe the relationship between PCD and bladder cancer. Clinical and pathological features were summarized according to previous reports and the present case (Table 3).

**Table 3:** Clinical and Pathological Features in Nine Cases of Bladder Cancer with PNS

Case No.	Author	Year	Age/Gender	Stage	Pathology	Clinical syndromes	Antibody	Treatment	Prognosis
1	Gita et al.[17]	2015	73/F	M^a^	Poorly differentiated carcinoma with squamous features	PEM	Negtive	Surgical resection and immunosuppression	Partial improvement
2	Syuichi et al.[12]	2013	66/M	N/A	Urothelial carcinoma	PCD	Anti-CKB	TURBT	No improvement
3	Lukacs et al.[20]	2012	76/F	pTa	Urothelial carcinoma	PEM and SSN	Anti-Hu	TURBT	Partial improvement
4	Forte et al.[21]	2009	76/M	pT2	High grade urothelial carcinoma	Neuromyotonia	Anti-VGKC	Resectionofthetumour	Complete improvement
5	Sean et al.[14]	2003	59/M	N/A	N/A	POM	Anti-Ri	N/A	N/A
6	Charles et al.[22]	2001	57/M	pT3	High grade urothelial carcinoma	POM	Anti-Ri	Radical cystectomy and immunosuppression	Partial improvement
7	Lowe et al.[23]	1992	71/M	pT3	High grade urothelial carcinoma	Visual changes, glossal spasm and dysphagia	N/A	Combination chemotherapy	Complete improvement
8	John et al.[11]	1999	64/F	pT2	High grade urothelial carcinoma	PCD	Anti-Yo	Partial resection of the bladder	No improvement
9	Current report	2015	68/M	pCIS	High grade urothelial carcinoma and the well-differentiated squamous cell carcinoma	PCD	Negtive	Laparoscopic radical cystectomy	Complete improvement

^a^A recurrent pelvis mass proven as urothelial carcinoma 22 years after radical cystectomy; *PEM* Paraneoplastic encephalomyelitis; *PCD* Paraneoplastic cerebellar degeneration; *SSN* Subacute sensory neuronopathy; *POM* Paraneoplastic opsoclonus-myoclonus; *Anti-CKB* anti-creatine kinase, brain-type; *N/A* Not available

The male to female ratio of 2 to 1 in the nine related cases is different from the bladder cancer incidence rate. The worldwide incidence rate is believed to be 8.9/100,000 and 2.2/100,000 for men and women, respectively. Therefore, the male to female ratio is more than 4 to 1 [1]. Sean et al. reported that the female to male ratio of 34 PNS patients seropositive for anti-Ri was 2 to 1 [14]. British data showed a striking female preponderance of PNS, with a female to male ratio of 2.3 to 1 [15]. Therefore, the female patients with bladder cancer may be predisposed to PNS. This finding is the same as for PNS caused by other cancers.

PNS is caused by high-grade muscle-invasive bladder cancer in most cases. Blood supplies of the muscle-invasive bladder cancer are richer, facilitating the circulation of neuronal antigens expressed by high-grade cancers. In the present case, the patient with the urothelial CIS and squamous cell carcinoma of the bladder suffered from PCD. Matsumoto L. reported that CIS of the testis triggered severe hypokinesis as a paraneoplastic manifestation and detection of anti-Ma2 antibodies [16]. In this case, the bladder cancers comprised high-grade urothelial carcinoma and well-differentiated squamous cell carcinoma. However, methods of verifying which type of carcinoma causing PCD are limited. Gita reported that recurrent bladder cancers with squamous features caused paraneoplastic encephalomyelitis [17]. Numerous reports of other organs have also demonstrated that PNS was caused by squamous cell carcinoma [18, 19]. Therefore, we believe the PNS is more likely to arise in patients with high grade urothelial carcinoma with squamous differentiation.

Onconeural antibodies are of vital importance in PNS pathogenesis and diagnosis. However, only 60–70 % of patients have detectable onconeural antibodies [10]. Onconeural antibodies were not detected in the present case. Table 3 shows that the bladder cancer antibodies inducing PNS are anti-Ri, anti-Hu, anti-Yo and anti-VGKC. SCC and CYFRA21-1 serum concentrations were increased. SCC and CYFRA21-1 are squamous cell carcinoma markers and not paraneoplastic antibodies. No relationship between the low serum thyroglobulin level and PCD is believed to exist. The low serum thyroglobulin level may be due to primary thyroid changes.

Early treatment of the related tumour is the best way to promote neurological improvement. The therapeutic effects of immune therapy were unsatisfactory in these nine cases. The underlying bladder cancer received early diagnosis and timely radical cystectomy. Therefore, injury to the nervous systems was reversible, and PNS disappeared in the present case. Thus, patients with muscle-invasive bladder cancer or CIS of the bladder that simultaneously suffered from PNS require timely radical cystectomy to avoid irreversible injury to the nervous system.

## Conclusion

The patient in the present case suffering from PCD attained a good prognosis. This prognosis was due to early diagnosis and timely radical cystectomy. PNS has an obvious female preponderance in patients with bladder cancers. PNS is more likely to arise in patients with high grade urothelial carcinoma with squamous differentiation. Therefore, timely radical cystectomy is necessary to achieve a good prognosis.

### Ethics approval and consent to participate

The study was approved by the Ethics Committee of Qilu Hospital of Shandong University and the methods were carried out in accordance with the approved guidelines.

### Consent for publication

Written informed consent was obtained from the patient for publication of this case report and any accompanying images. A copy of the written consent is available for review by the Editor of this journal.

## Abbreviations

PNS: paraneoplastic neurological syndromes
PCD: paraneoplastic cerebellar degeneration
TURBt: transurethral resection of bladder tumor
CIS: Carcinoma *in situ*
BCG: Bacillus Calmette-Guérin
RC: radical cystectomies
CSF: cerebrospinal fluid
MRI: magnetic resonance imaging
CT: computed tomography
